# Temporal Weighted Averaging for Asynchronous Federated Intrusion Detection Systems

**DOI:** 10.1155/2021/5844728

**Published:** 2021-12-17

**Authors:** Shaashwat Agrawal, Aditi Chowdhuri, Sagnik Sarkar, Ramani Selvanambi, Thippa Reddy Gadekallu

**Affiliations:** ^1^School of Computer Science and Engineering, Vellore Institute of Technology, Vellore, India; ^2^School of Information Technology and Engineering, Vellore Institute of Technology, Vellore, India

## Abstract

Federated learning (FL) is an emerging subdomain of machine learning (ML) in a distributed and heterogeneous setup. It provides efficient training architecture, sufficient data, and privacy-preserving communication for boosting the performance and feasibility of ML algorithms. In this environment, the resultant global model produced by averaging various trained client models is vital. During each round of FL, model parameters are transferred from each client device to the server while the server waits for all models before it can average them. In a realistic scenario, waiting for all clients to communicate their model parameters, where client models are trained on low-power Internet of Things (IoT) devices, can result in a deadlock. In this paper, a novel temporal model averaging algorithm is proposed for asynchronous federated learning (AFL). Our approach uses a dynamic expectation function that computes the number of client models expected in each round and a weighted averaging algorithm for continuous modification of the global model. This ensures that the federated architecture is not stuck in a deadlock all the while increasing the throughput of the server and clients. To implicate the importance of asynchronicity in cybersecurity, the proposed algorithm is tested using NSL-KDD intrusion detection system datasets. The performance accuracy of the global model is about 99.5% on the dataset, outperforming traditional FL models in anomaly detection. In terms of asynchronicity, we get an increased throughput of almost 10.17% for every 30 timesteps.

## 1. Introduction

**Figure 1:**
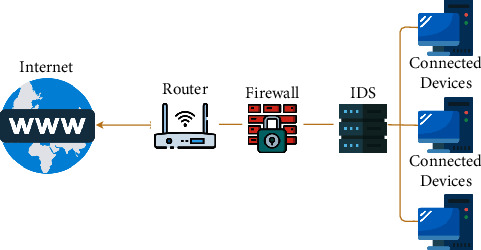
Architecture of network-based intrusion detection system (NIDS).

Digitization of information and currency has brought great simplicity to our lives. At the cost of this modernization, these digital assets (i.e., information, digital money, and intellectual property) are continuously in danger from cyberattacks and intrusions. To protect the privacy, confidentiality, and availability of these assets, protective security layers like firewalls, intrusion detection systems (IDSs), and, most importantly, security protocols are being used. Among these layers, IDSs have been researched extensively in recent years. Their contribution to the field of cybersecurity is immense.  Figure 1 shows a general structure of the of IDS deployment. On the one hand, a carefully selected and optimized IDS can prevent networks from even unknown threats [1]. On the other hand, a poorly selected IDS cannot prevent or classify unknown traffic and alert frequent false alarms. In this context, IDS can be divided into two major types: protocol-based intrusion detection systems (PIDSs) and anomaly-based intrusion detection systems (AIDSs).

AIDS [2] uses statistical models trained on existing intrusion detection data to analyze and classify incoming traffic. With the development of machine learning (ML) and deep learning (DL) algorithms and high availability of data, AIDS shows great promise. Usually, a DL-based IDS is trained on a centrally located dataset that is collected from various edge devices, servers, and computer networks. Works [3, 4] review the implementation of DL algorithms for various deployments of IDS: network intrusion detection systems (NIDSs), host intrusion detection systems (HIDSs), etc. Within DL, convolutional neural networks (CNNs), autoencoders [5], recurrent neural networks (RNNs) [6], generative adversarial networks (GANs) [7], and other architectures have been used to train intrusion detection systems. The variety in architectures comes from the different datasets that are available for the training of IDS: NSL-KDD dataset [8], CICIDS dataset [9], Bot-IoT dataset [10], etc. The state-of-the-art DL models have achieved 98–99% accuracy in most available datasets. Despite their success, DL models lose their feasibility in realistic scenarios. In the deployment of IDS in a healthcare center, the data cannot simply be transported to the central location since it can easily sniffed. Also, the latency caused by data transmission can reduce the efficiency of IDS and introduce a network latency. To overcome the limitations of DL, a new privacy-preserving ML architecture called federated learning (FL) [11] is employed for IDS.

FL [12] provides a collaborative learning setup where client edge devices train ML/DL models based on their own local data and the updated models are aggregated to form a single global model. This iteration is repeated until a required output for the application is attained. This architecture of FL is suitable for the deployment of anomaly-based IDS [13], especially in vulnerable environments where cybersecurity is a must [14–16]. The involvement of heterogeneous clients and abundance in data result in IDS with high detection and low false alarm rate. It also promotes edge computation [17], making the system more efficient to intrusions from all directions. Even the implementation of conventional FL is vulnerable to high communication overheads and high latency. Consider a federated environment with 1000 clients and 1 server. With varying parameters of edge devices such as computational power, network bandwidth, and so on, time taken for each device to train the models and transmit these parameters would differ. In some cases, due to failed transmissions, the server may end up in a deadlock. AFL [18, 19] has been proposed to tackle this problem. It ensures continuous modification of the global model, irrespective of the transmissions received from the clients.

In this paper, we propose a novel temporal weighted averaging algorithm for the implementation of AFL in IDS. The server waiting period for our AFL algorithm is described by an expectation function after which it continues with the next round of federated training. The algorithm also includes a weighted averaging algorithm that takes into account the round identity of a client. Temporal weighted averaging enables continuous communication and optimal throughput for server and clients. A simulation of network delays is executed based on model training times in an intrusion detection environment for better deployment of cybersecurity systems. The main aim of this study is the development of efficient IDS with high throughput and maximum performance accuracy. The main contributions of this paper are as follows:

1. An asynchronous federated learning algorithm for the implementation of efficient intrusion detection systems. The proposed algorithm utilizes a novel temporal weighted averaging methodology for modifying the global client.
2. An expectation function is defined that determines the waiting time of the server after which a new round of broadcast is initiated.
3. Experiments are conducted on an intrusion detection environment with the help of the NSL-KDD dataset. The computation differences and communication lags are simulated during the training of the client models, and the performance of the algorithm is measured in terms of time complexity and accuracy obtained.

The structure of the paper is as follows.  Section 2 reviews the domains of DL, FL, and AFL by highlighting their importance in the implementation of IDS and certain limitations.  Section 3 provides a detailed explanation of the proposed algorithm. In Section 4, the performance evaluation of the algorithm is conducted, and Section 5 concludes the paper.

## 2. Literature Survey

In this section, a survey of the state-of-the-art DL and FL algorithms for the implementation of IDS is performed. Certain limitations of conventional DL algorithms as well as FL architectures are identified. To overcome some of these limitations, asynchronous FL is reviewed. The focus is on the depiction of federated system latency and deadlocks caused by various communication and computation factors.

### 2.1. Deep Learning-Based IDS

In recent years, the research on anomaly-based IDS using deep learning has increased [20, 21]. J48 trees, multilayer perceptron (MLP), and Bayes network are algorithms used in [22]. These algorithms tackle the issue of low accuracy when IDS uses ANN with fuzzy clustering to detect uncommon attacks. To improve the accuracy, the method proposed in [22] splits the heterogeneous set of training data into homogeneous subsets which reduces the complexity of the data. The major drawback in these algorithms was that they were unable to apply feature selection to restore valuable features and cut out redundant and unwanted features. Bhavani et al. [23] used a random forest classifier on the NSL-KDD dataset to obtain an accuracy of 95.323%. Smart feature selection that uses Gini importance has been utilized in [23] to reduce the number of features, thereby reducing the complexity of the model. The downside of this classifier is that it poses an issue of not detecting low-frequency attacks and high false-positive rates. An occasional low-frequency attack might pose a threat to the network leaving the system vulnerable. These false-positive rates can become a nuisance while deploying the model in a real-time setting. An ensemble-based learning method [24] along with K-means, K-nearest neighbors, fuzzy C-means, Naïve Bayes, support vector machine, and radial basis function algorithms was used based on the six algorithms for network traffic anomaly detection in [25]. In [25], the results of innumerable supervised and unsupervised algorithms were clustered using voting. It has amplified the accuracy and performance of the current IDS. Here the pitfall is that recall is low in a few cases that stipulate a high value of false-negative rate. XGBoost [26] and AdaBoost with and without clustering were used in [27] to train a model for network intrusion detection. Verma et al. [27] trained the model on the NSL-KDD dataset. An accuracy of 84.253% was obtained. The issue here is that there is still room for improvement in the performance which can be done by utilizing hybrid or ensemble machine learning classifiers. The false-positive rates can be minimized which would ultimately lead to improvement in accuracy and precision. Multiple ML models were implemented using various ML algorithms on the NSL-KDD dataset in [28]. They deploy feature selection using the wrapper method that helps improve the accuracy as compared to the other works on the same dataset. The work done in [28] focuses only on signature-based attacks, thereby leaving novel attacks undetected which is a major setback; another issue is that the new attacks or zero-day attack detection remains unsolved due to the high false-positive rate [28]. Ganapathy et al. [29] proposed an intelligent conditional random field (CRF)-based feature selection algorithm to optimize the number of features. Furthermore, a layered approach-based algorithm was used to perform classification on these reduced features.

### 2.2. Federated Learning-Based IDS

Over the last few years, federated learning has been endorsed for addressing numeral issues in applied ML. The application of ML is limited to a local system, thus restraining its scalability. Ml has data stored centrally that also affects the privacy of the data and makes it vulnerable to attacks. FL is more scalable and can be deployed on a network and can be personalized according to the system requirement individually. FL has decentralized data, and rather than exchanging data, models are used for communication. This makes it more secure and helps in preserving the privacy. In addition, the difference between FL-based IDS and ML-based IDS can be observed in Table 1.

Federated learning is an emerging artificial intelligence technology. The work proposed in [30] talks about a permission-based federated learning method to obtain an anomaly detection model. Here the contributing parties in the federated learning can be held accountable and have their model updates audited. The drawback in [30] is that the model gets along with heterogeneous hardware and fails to cope with unreliable network connectivity and intermittently connected nodes. An autonomous self-learning distributed system for detecting compromised IoT devices was designed in [31] using federated learning for intrusion detection. But the setback in [31] was that it failed to detect poisoning attacks. Chen et al. [32] proposed a federated deep autoencoding Gaussian mixture model (FDAGMM) that performs network anomaly detection tasks better than the traditional deep autoencoding Gaussian mixture model (DAGMM) due to the availability of limited data. The weakness in the model given in [32] is that it can work only on data records that have the same feature structure, thus making it less versatile to be deployed on other application domains. A multitask deep neural network in federated learning (MT-DNN-FL) is presented in [33] to perform network anomaly detection tasks. It offers simultaneous execution of tasks like network anomaly detection, VPN (Tor) traffic recognition, and traffic classification, which provides more information to the network administrator. However, the training performance in [33] is considerably low.

### 2.3. Asynchronous Federated Learning

A privacy-preserving asynchronous federated learning mechanism for edge network computing (PAFLM) was proposed in [34] that allows the node to join or quit in any process of learning, and this makes it suitable for highly mobile edge devices. This allows multiple edge nodes to obtain more coherent federated learning without the fuss of sharing private data. Chen et al. [18] proposed an asynchronous federated learning framework; here the distributed clients with continuously arriving data learn from an efficiently shared model collaboratively. ASO-fed is computationally more effective than synchronized FL as ASO-fed need not wait for other clients to carry out gradient updates. Chen et al. [35] proposed an enhanced federated learning technique by deploying an asynchronous learning approach on the clients and temporally weighted aggregation of the local models on the server.

These conclusions from the existing literature have helped in acknowledging that the best way to go about this problem is to use asynchronous federated learning model as it makes the process more efficient and offers better performance in intrusion detection. Deep learning-based intrusion detection systems are generally limited to the local system constraining the range and scale of the implementation. However, federated learning models are scalable and can be deployed in a network as an effective intrusion detection system. This system however is plagued by client delay and excessive server idle time [36]. Asynchronous federated learning combats high server idle time by eliminating the wait parameter and aggregates the model responses on the go. This however creates ambiguity in the importance of a client response based on the round in which it originated. This limitation is tackled by the proposed temporal weighted averaging algorithm.

## 3. Temporal Weighted Averaging Algorithm

**Figure 2:**
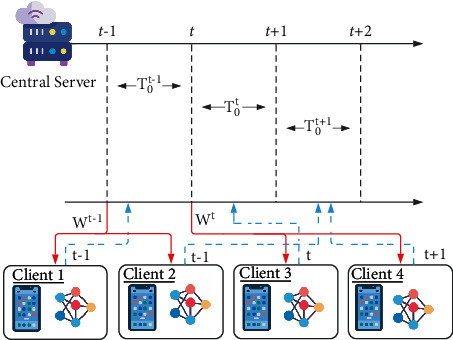
Proposed asynchronous federated algorithm.

In this section, we provide a detailed description and flow of the temporal weighted averaging algorithm. The complete algorithm can be observed in Figure 2. The novelty in implementation can be divided into 2 main formulations: expectation function for idle time calculation and temporal weighted average for AFL. Various characteristics of the formulation are discussed in this section followed by experimental results on several hyperparameters: client ratio, learning rate (*η*), initial threshold time (*C*), etc. The client and server procedures that are proposed are formulated in Algorithm 1.

In a regular federated learning framework, the server initializes the weights for the server. After this step, the entire framework undergoes a series of steps in cycles called rounds. These rounds consist of three broad phases—server weight broadcast, client model training, and aggregation of client weight updates. In the server weight broadcast phase, the weights of the server model are broadcast to each and every client device. These weights are received by the clients in their receive buffer. For the next phase, the client device trains the received model and trains on the local data. The weight update obtained from the training of the client models is sent back to the server and accumulated to the server weights in the aggregation phase. A standard measure of central tendency like mean (equation (1)) is used for the aggregation of the client weight updates in the server.

(1) $$\begin{array}{c} w^{\prime }=\frac{\sum_{i=0}^{r}w_{i}}{\sum_{i=0}^{r}i}, \end{array}$$

where *C* is the initial waiting time that is assigned in the beginning based on the observed training times, model complexity, and average communication cost. Although *C* is not chosen randomly, the phasing is such that it does not drastically effect the algorithm. This is because real-time environments are unpredictable and certain parameters cannot always be measured. After round 0, its value is modified dynamically by an expectation function *F*(*r*) as shown in the following equation:

(2) $$\begin{array}{c} F\left(r\right)=P\left(e\right)-P\left(r\right), \end{array}$$

(3) $$\begin{array}{c} \Rightarrow F\left(r\right)=\frac{e-r}{n}, \end{array}$$

where *P*(*x*) represents the ratio of *x* clients being received out of *n*. So, if *e* is expected to be 8 in a 10-client setup, then *P*(*e*) = 0.8. In each round, if fewer clients are able to transmit their data than the expected (*e*), the value of *P*(*e*) becomes positive while if more are transmitted, then *P*(*e*) becomes negative. This expectation function defines the waiting time of the central server. Initially, the threshold waiting time (*T*_0_) for which the server waits before aggregation is equal to *C*. After the 1^*st*^ round, this value is calculated using the following equation:

(4) $$\begin{array}{c} T_{0}=T_{0}+\frac{e-r}{n}. \end{array}$$

This equation is not directly dependent on edge device parameters of communication costs but still ensures the stability of waiting to maximize throughput. Its adaptability comes solely from the number of clients received keeping it simple yet effective.

The aggregation of the number of client weight updates that have been received takes place at the end of each timestep. During this aggregation phase, the client models encountered belong to weight updates originating in various timesteps. This difference in the origin of timesteps needs to be addressed to make sure that the global model addresses the importance of the latest updates and does not lag behind and perform poorly on time-sensitive data. To instigate attention in this direction, usage of (5) for aggregation of the client weight updates is proposed. Here, *w*′, *R*_*i*_, and *w*_*i*_ stand for weight update for the server model, the timestep in which the weight update originates, and the weight update procured from the client device, respectively.

(5) $$\begin{array}{c} w^{\prime }=\frac{\sum_{i=0}^{r}R_{i}w_{i}}{\sum_{i=0}^{r}R_{i}}. \end{array}$$

Equation (5) used for aggregation is an elementary measure of central tendency, weighted mean. However, in this case, the weights for the weighted average are replaced by the origin timestep for the weight update. The fraction *R*_*i*_/∑*R*_*i*_ represents the ratio in which the models contribute to the weight update. Therefore, the higher or more recent the timestep is, the greater contribution the weight updates have in the overall learning of the server model.

## 4. Results and Discussion

This section deals with preprocessing of the data, experimental results, and the analysis of the resulting observations. The Dataset and Data Preprocessing section describes the dataset and the preprocessing steps for the data. The Model Architecture section discusses the architecture of the model used and its various parameters followed by the Training and Testing section which focuses on the training paradigm. Finally, the analysis of the trends and performance of the proposed architecture in comparison with generic federated learning architecture and other recent literature is shown in Performance Evaluation section.

For testing, implementation, and simulation purposes, Python 3.8.10 on a Ubuntu 20.04.2 LTS local machine has been used. TensorFlow 2.4.0 has been majorly used as a deep learning framework in tandem with CUDA 11.0 and cuDNN 8.1.0 running on an NVIDIA GeForce RTX 2070 GPU.

### 4.1. Dataset and Data Preprocessing

The dataset used in the implementation of the federated learning framework is the NSL-KDD dataset. The NSL-KDD dataset was generated by the Canadian Institute for Cybersecurity. This dataset has 43 columns and 125 973 records of data. The data in the columns are both categorical and continuous data; therefore, preprocessing of the data needs to be customized for every column based on the nature of data. The categorical data like the type of protocol used (FTP, HTTP, SMTP, etc.) and nature of connection (TCP, UDP, etc.) are encoded to an encoding vector representing the class to which the record belongs. The categorical data are then replaced by the encoding vector. The data column that corresponds to the continuous data is Min-Max scaled with (6). This reduces the range of the continuous data to [0,1] and makes the data compatible with the neurons. The final number of columns for the dataset after all the preprocessing is 123. The increase in the number of columns is attributed to the role of the encoding vectors.

(6) $$\begin{array}{c} x^{\prime }=\frac{x-\min\left(x\right)}{\max\left(x\right)-\min\left(x\right)}. \end{array}$$

**Figure 3:**
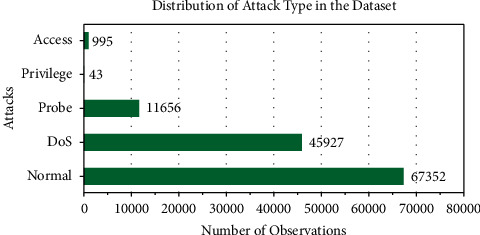
Distribution of the types of attacks in the NSL-KDD dataset.

The target observations are either an attack label or normal. The attack labels are grouped into the following types of attacks: access, privilege, probe, and DoS, to reduce the target size of the IDS. The high target size of IDS can lead to more focus being put on recognizing the attack itself rather than classifying whether there is an abnormality in the packet. After grouping the attacks, the distribution of the labels in the dataset is as shown in Figure 3. This level of abstraction in the data is optimal for an IDS to detect an attack and provide brief information about the type of attack. This information can help in customizing the action taken against the anomalous packets.

### 4.2. Model Architecture

**Figure 4:**
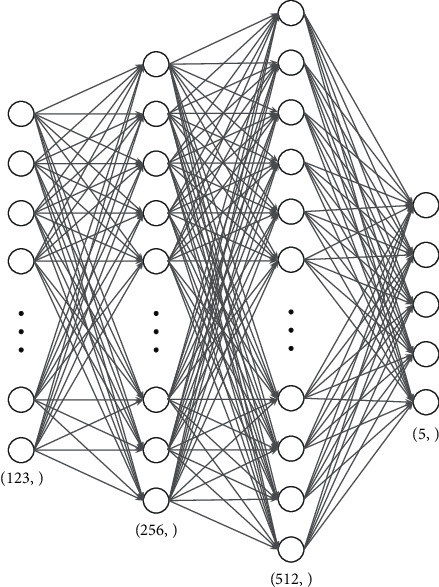
Model architecture for the global model and the client models.

The model used for the client and server models is a four-layer deep neural network as shown in Figure 4. A small and light model is used in this scenario as the IDS. This is due to the fact that a smaller model implements fewer number of parameters, thus reducing the amount of information that needs to be communicated during the server model broadcast and the client model aggregation phases. Furthermore, a lighter model is faster to train and takes up less amount of energy and demands less computation power in edge devices. The input layer is a 123-neuron wide layer that corresponds to each input parameter of the dataset. This input is connected to a hidden layer of size of 265 neurons. The output of this layer is connected to the next hidden layer of size of 512 neurons. This layer is finally connected to an output layer of size of 5 neurons corresponding to each type of attack as mentioned in Section 4.1. The model is trained with Adam optimizer with cross-entropy as the loss function.

### 4.3. Training and Testing

The server model is initialized with random weights close to zero. The constant *T*_0_ is set to 4 seconds based on a short preliminary run to determine the approximate time taken for broadcast, training of client model, and aggregation. This provides a good starting point for the time threshold. This threshold value adapts to the situation based on the number of client models aggregated. Additionally, it makes sure that the server does not wait indefinitely for an expected response from client devices while making sure that a decent number of clients get aggregated at a single timestep. This threshold also prevents huge idle times for the server while it is waiting for the response of the clients. In this particular experimental setup, we have used 30 clients running for 15 rounds/timestep for the generic FL and the proposed architecture.

**Figure 5:**
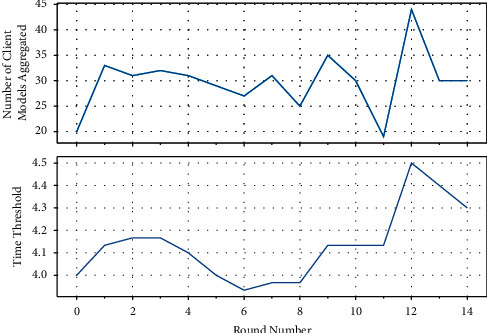
Number of client models aggregated (top) per timestep and its corresponding time threshold (*T*_0_) (bottom).

Once the server broadcasts the weights of the server model, it starts a timer for the threshold value. Once the time threshold is reached, the server aggregates all the models in the receive buffer. The value of *T*_0_ is also updated based on equation (4). The server then rebroadcasts the server model to the clients and starts the timer for the new threshold value. This process is repeated for the required number of timesteps. In deployment, however, this system can be run perpetually due to the adaptive nature of the threshold function. Due to the adaptive nature of the function, the number of models aggregated per timestep tends to hover about a certain range in time as shown in Figure 5.

It can also be observed in Figure 5 that the time threshold tends to stagnate when the amount of client models aggregated meets the expectation. However, when the number of clients aggregated falls short of the expectation value (like in the 11^*th*^ timestep), function 4 automatically corrects for this anomaly and increases the time threshold (as observed in the 12^*th*^ timestep). Similarly, when the server wait time is so high that it aggregates a lot of client weight updates, the time threshold falls and brings this exceeding response to the expectation level (this behaviour can be observed in the 12^*th*^ and 13^*th*^ timesteps). The system automatically corrects for both positive and negative deviations. These corrections of the deviations are independent of the time scale.

**Figure 6:**
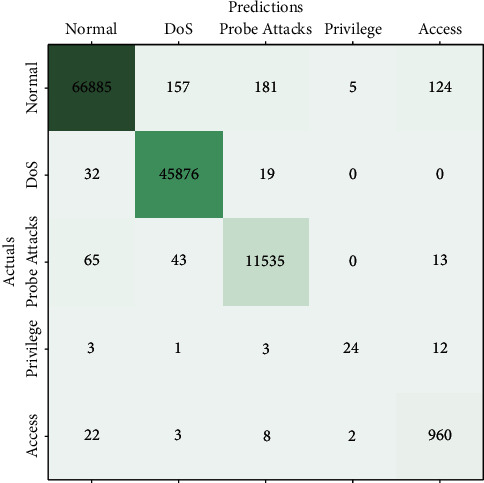
Confusion matrix for the server model after 15 rounds.

The weights of the models are aggregated in the ratio of the timestep in which the weights have originated. This makes sure that higher importance is given to the data aggregated in the recent timesteps over the ones that have originated significant timesteps back. Due to the decrease in the idle time of the server, the federated architecture implementing the temporal weighted averaging converges comparatively faster.  Figure 6 depicts the prediction versus actual labels for the attacks. It is evident that the probe attacks are similar in characteristics to benign network packets. This behaviour is prominent from the high mislabeled packets in the confusion matrix.

### 4.4. Performance Evaluation

Temporal weighted averaging is tested using the NSL-KDD dataset with a federated and non-IID setup. The performance of the algorithm is measured in terms of prediction accuracy, throughput of the server, and, most importantly, idleness of the system as a whole.

**Figure 7:**
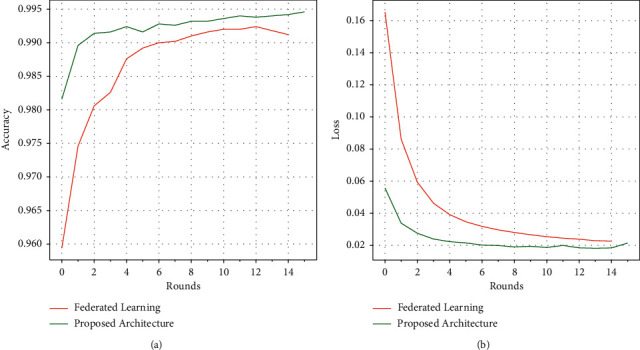
Comparison of the performance of the federated learning architectures. (a) The plot of the accuracy of the global model in each round. (b) The loss scores of the global model for each round.

For the evaluation of prediction capability, validation accuracy is used.  Figure 7 shows the achieved accuracy and loss values after training for 15 timesteps on a population of 30 clients. An accuracy of 99.46% is achieved on the data that is at par with some of the recent works in the domain of intrusion detection. Statistical comparison between the proposed algorithm and other recent works is shown in Table 2. This is accomplished despite the fact that less ratio of clients contributes to the aggregated global model per round.

In terms of time complexity, we achieve a 10.17% increase in throughput as compared to the generic federated averaging algorithm. This results in better time utility of the server and removes almost any possibilities of deadlock. For training of 30 clients for 30 rounds, generic FL takes about 69.1 s where the temporal weighted average takes approximately 62 s. Not only training but also usage of the lightweight model decreases the prediction time in edge devices. In a more realistic environment, a 10% increase in throughput especially in IDS applications would affect the network greatly. Although asynchronous algorithms greatly improve performance, in rare instances, they can introduce certain discrepancies that can hamper the composure of the system. Lack of cohesion in asynchronous algorithms can result in overlooking of certain traffic or even misinterpretation of their nature. Other risks include reduced rest time for the server, increased chances of overloading, and uneven response accuracy. Temporal weighted averaging also considers some of these risks by providing a work-rest trade-off.

## 5. Conclusion

In this paper, the main objective is to implement temporal weighted averaging for asynchronous federated learning. This algorithm utilizes a novel temporal weighted averaging methodology for modifying global clients. This architecture is deployed in contrast to traditional federated learning algorithms that are highly constrained due to communication cost and latency. The proposed solution is to simulate an intrusion detection environment using the NSL-KDD dataset and perform experiments on the model. The resulting accuracy of 99.5% is at par with current state-of-the-art federated learning algorithms but easily surpasses them with respect to high system throughput.

Even though the current work has proven successful in its attempt to streamline the efficiency of intrusion detection systems, there is still more scope for improvement. The dataset used for experiments may be diversified to include more heterogeneous data. Other intrusion detection datasets can also be used in the future to validate the proposed algorithm. The number of parameters present in a federated architecture is huge, and fine-tuning each is not feasible. More work can be done by optimizing the parameters efficiently.

## Figures and Tables

**Algorithm 1:**
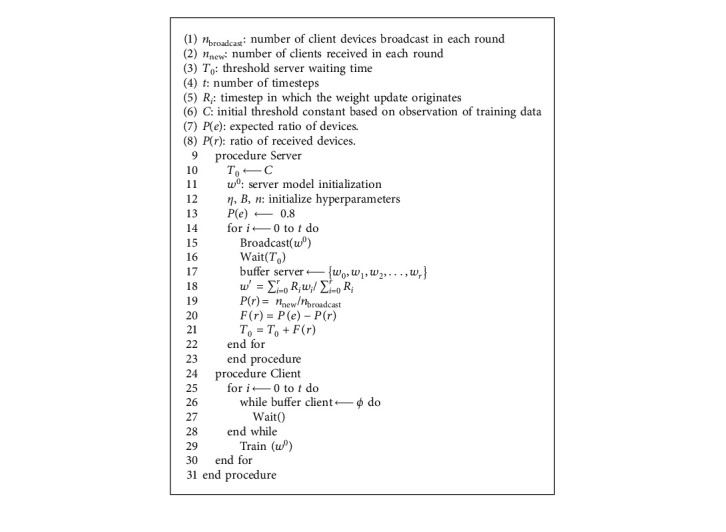
Algorithm 1Temporal weighted averaging.

**Table 1:** Summary of DL-based IDS and FL-based IDS.

Sl. No.	DL-based IDS	FL-based IDS
1	Constrained to a local system.	Scalable and readily deployable in a network.
2	Pretrained or active training has limited relationship with similar nodes in the network.	Extensive relationship between models. Promotes general system learning and personalization for each node.
3	Less effective for evolving intrusions and response to novel attacks.	Structured to learn from every node's individual experience and respond to unforeseen attacks.

**Table 2:** Performance comparison of multiple architectures.

Sl. No.	Algorithm	Clients	Rounds/timesteps	Accuracy
1	Asynchronous FL with temporal weighted averaging	30	15	99.459
2	Federated learning	30	15	99.11
3	FTML (federated teacher mimic learning) [37]	10	20	98.118
4	FSML (federated student mimic learning) [37]	10	20	98.110
5	Collaborative anomaly detection [38]	4	4	98.24
6	PHEC in federated setup [39]	4	Adaptive	88.42

## Data Availability

The dataset used in this work, “NSL-KDD dataset,” is available at https://www.unb.ca/cic/datasets/nsl.html.
